# Comparison of CMR DENSE strain imaging and 2D speckle tracking echocardiography for cardiac resynchronization therapy (CRT) response

**DOI:** 10.1186/1532-429X-18-S1-O126

**Published:** 2016-01-27

**Authors:** Jorge A Gonzalez, Daniel A Auger, Xiao Chen, Michael Salerno, Christopher M Kramer, Frederick H Epstein, Kenneth C Bilchick

**Affiliations:** 1 grid.27755.32000000009136933XCardiology, University of Virginia, Charlottesville, VA USA; 2 grid.27755.32000000009136933XBiomedical Engineering, University of Virginia, Charlottesville, VA USA

## Background

Considering that nonresponse rates to cardiac resynchronization therapy (CRT) are typically 35-50%, assessment of the cardiac substrate for CRT with cardiac imaging could be very helpful for many patients with heart failure referred for this therapy. We have previously shown that CMR assessment of dyssynchrony using displacement encoding with stimulated echoes (DENSE) is strongly associated with CRT response; however, there has not yet been a direct comparison between this CMR methodology and more commonly used 2D speckle tracking echocardiography (STE) dyssynchrony parameters.

## Methods

We imaged 60 patients with CMR DENSE and 2D STE. STE was performed using commercial ultrasound by acquiring digital routine greyscale 2-D cine loops from 3 consecutive beats obtained at the end of expiration from standard apical views (2,3 and 4-chamber) and in basal and mid-cavity short-axis slices. Differences in time to peak (TTP) radial, transverse and longitudinal strain in opposing walls were determined using standard methods. CMR spiral cine DENSE was performed using a 1.5 T MR scanner. CMR dyssynchrony was determined using the circumferential uniformity estimate with singular value decomposition (CURE-SVD), as we have previously described. We examined associations between these dyssynchrony measures and the extent of LV reverse remodeling based on the change in LV end-systolic volume (LVESV) 6 months after CRT. Linear regression analysis was performed using SAS 9.4.

## Results

**Figure 1:**
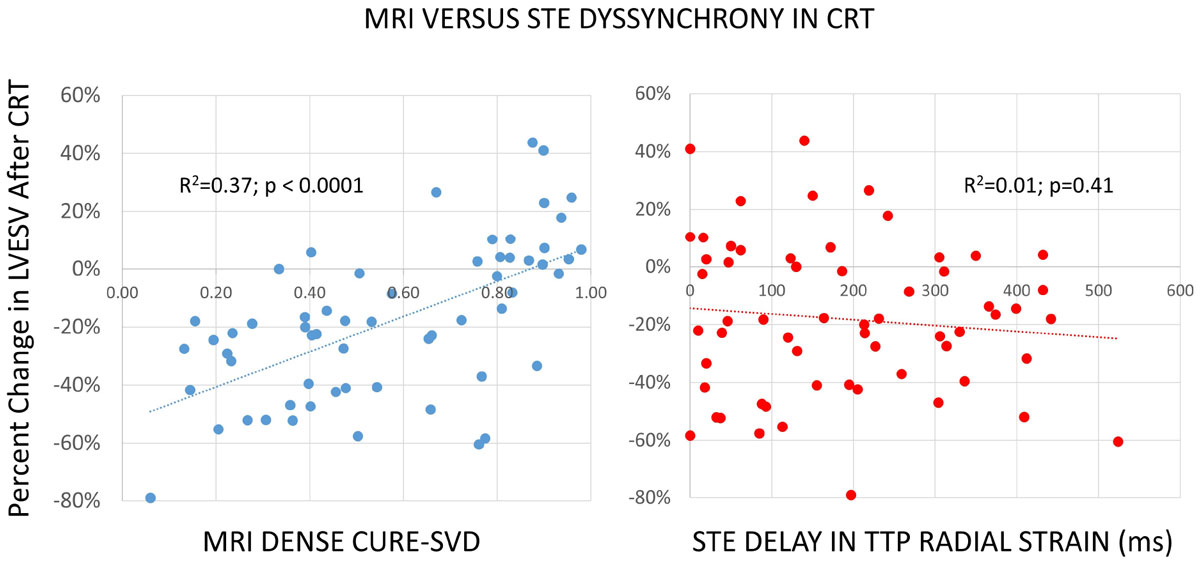
**MRI DENSE CURE-SVD versus STE dyssynchrony in CRT**.

The cohort was 33.3% female with a median age of 64.9 years (IQR 57.8-72.0 years), QRS duration of 156 ms (IQR 147-177 ms), and LV ejection fraction (LVEF) of 0.23 (IQR 0.16-0.28). The overall response rate based on LVRR was 58%. CURE-SVD with MRI cine DENSE was strongly associated with the degree of LVRR with an R^2^ of 0.37 (p < 0.0001), whereas none of the STE parameters had significant linear associations with the degree of LVRR after CRT. As shown in Figure 1, the most commonly used 2D STE parameter - the delay in time to peak radial strain in opposing anteroseptal and posterolateral walls (STE radial delay) - was not associated with the degree of LVRR after CRT (R^2^ = 0.01; p = 0.41). Furthermore, the relationship between CURE-SVD and the STE radial delay was quite weak (R^2^ = 0.005; p = 0.61). The R^2^ values for the relationships between the degree of LVRR after CRT and the maximal time delay in TTP transverse or longitudinal strain in opposing walls from apical long-axis views were also not significant (transverse R^2^ = 0.04 [p = 0.13]; longitudinal R^2^ = 0.02 [p = 0.25]).

## Conclusions

CMR assessment of dyssynchrony with CMR DENSE CURE-SVD is strongly associated with response to CRT based on LVRR criteria, but STE parameters were not associated with the extent of LVRR after CRT in this cohort. Furthermore, there were minimal associations between STE delays in TTP strain and CURE-SVD determined with CMR DENSE. Thus, CMR strain analysis is superior to STE for assessment of the likelihood of CRT response.

